# Waldenström macroglobulinemia with secondary pure red cell aplasia in a patient with metastatic castrate resistant prostate cancer receiving an immune checkpoint inhibitor: a case report

**DOI:** 10.1186/s13256-023-03948-4

**Published:** 2023-05-28

**Authors:** Vaibhav Kumar, Nathan D. Montgomery, Hendrik W. van Deventer, Young E. Whang

**Affiliations:** 1 Division of Hematology, Department of Medicine, University of North Carolina at Chapel Hill, Chapel Hill, NC USA; 2 Division of Oncology, Department of Medicine, University of North Carolina at Chapel Hill, 170 Manning Drive, Chapel Hill, NC 27599-7305 USA; 3 Department of Pathology and Laboratory Medicine, University of North Carolina at Chapel Hill, Chapel Hill, NC USA

**Keywords:** Prostate cancer, Immune checkpoint inhibitors, Waldenström macroglobulinemia, Pure red cell aplasia, Case report

## Abstract

**Background:**

Hypoproliferative anemia is a frequently encountered adverse event in cancer patients receiving immune checkpoint inhibitors (ICI). Secondary pure red cell aplasia (PRCA) is a rare but recognized immune related adverse event. With the burgeoning use of ICIs, the association of secondary PRCA with an underlying lymphoproliferative disorder is often overlooked.

**Case presentation:**

We report a case of a 67-year-old non-Hispanic Caucasian male with metastatic castrate resistant prostate cancer, who developed severe transfusion dependent anemia with reticulocytopenia while receiving treatment with olaparib and pembrolizumab. His bone marrow findings demonstrated erythroid hypoplasia, in addition to a CD5-negative, CD10-negative monotypic B-cell population and a somatic *MYD88*L265P mutation. With a presence of an IgM-paraprotein, he was diagnosed with Waldenström macroglobulinemia (WM) with secondary PRCA and treated with 6 cycles of bendamustine and rituximab. He achieved a complete response with this regimen and was transfusion independent.

**Conclusion:**

In this case, underlying WM was uncovered through systematic investigation of anemia caused by ICI therapy. This report highlights the possibility of a lymphoproliferative disorder in patients with concerns for PRCA with prior ICI exposure. If identified, treating the underlying lymphoproliferative disorder is highly efficacious in the management of the secondary PRCA.

## Background

There are multiple possible etiologies for new onset anemia in patients with solid organ malignancies, ranging from marrow infiltration from underlying cancer, anemia of chronic inflammation, treatment related complications or (especially considering the demographics of the patients) a separate pathology distinct from the underlying disease [1]. As such, the diagnosis and management of new onset anemia in this patient population poses a clinical dilemma. This is particularly pertinent with the increasing use of immune checkpoint inhibitors (ICIs) either alone or in combinations with myelosuppressive agents such as poly-ADP-ribose polymerase (PARP) inhibitors [2]. Better understanding of the hematologic toxicity profile of these combinations is essential in providing therapeutic insights into appropriate use of these agents. Herein, we describe a case of new onset severe hypoproliferative anemia in a patient with metastatic castrate resistant prostate cancer (mCRPC) receiving combined PARP inhibitor and ICI.

## Case presentation

In May 2019, a 67-year-old Caucasian male demonstrated progression of his mCRPC. Repeat staging had revealed progression of his previously known hepatic metastasis and development of new sites of bone metastases. Germline mutation testing revealed a pathogenic variant of *BRCA2*. Prior systemic therapy for metastatic prostate cancer included bicalutamide (1 year), enzalutamide (3 years), sipuleucel-T (6 weeks), and leuprolide (6 months). He was started on the PARP inhibitor olaparib 300 mg twice daily. Based on the results of phase II trial by Karzai *et al.* [2], a checkpoint inhibitor was added, but pembrolizumab (200 mg intravenously every 3 weeks) was substituted for durvalumab due to insurance coverage.

**Fig. 1:**
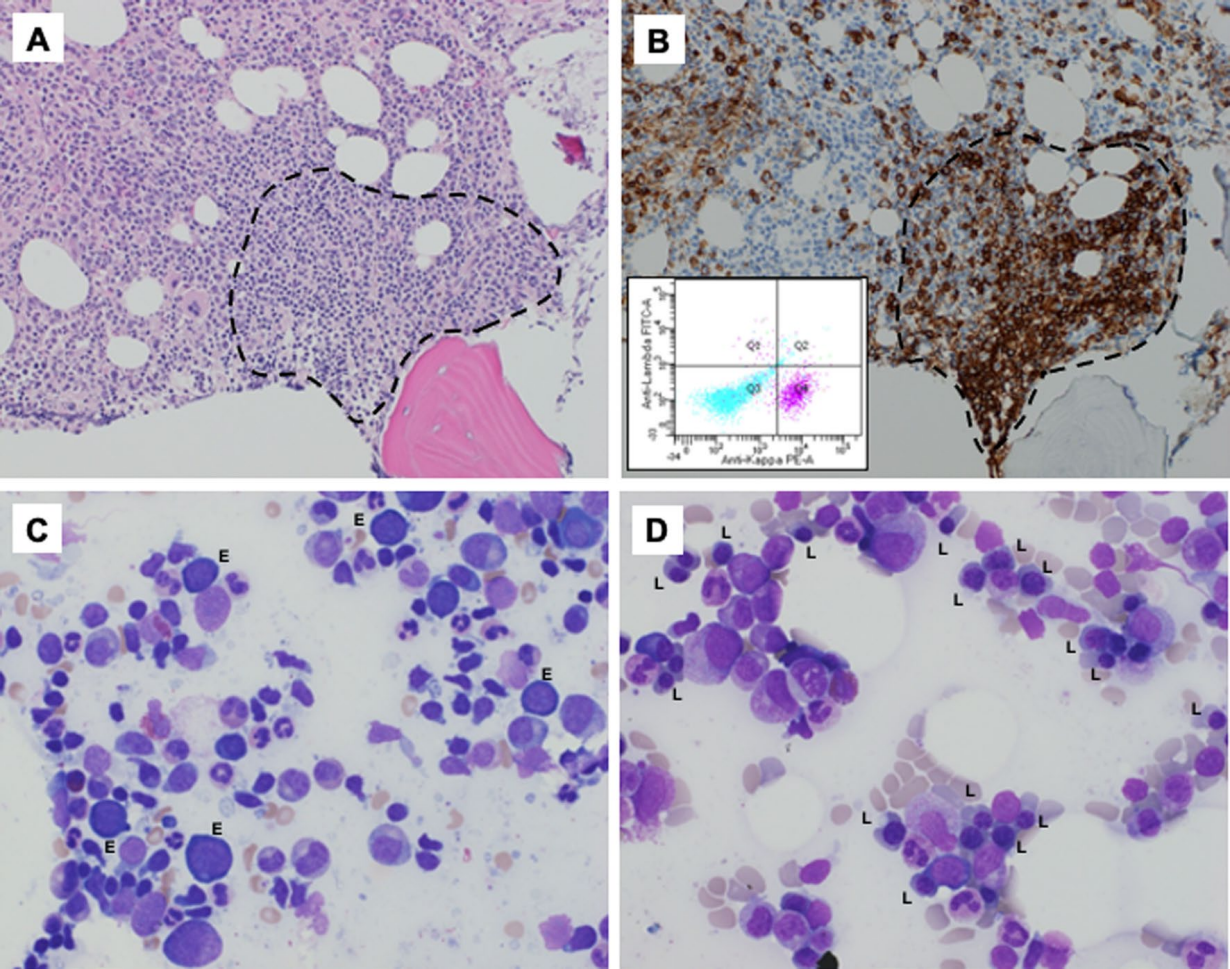
Initial and post treatment bone marrow evaluation. Bone marrow findings pre- (**A**–**C**) and post- (**D**) treatment. **A** The hematoxylin and eosin stained bone marrow core biopsy, ×20 objective magnification, shows a paratrabecular lymphoid aggregate (dashed line). **B** CD20 immunohistochemical stain, ×20 objective magnification, demonstrates that the lymphocytes in the aggregate are B-cell predominant (dashed line). The inset shows flow cytometry plots, gated on lymphocytes. T cells are shown in aqua, and B cells are shown in magenta. The B cells are uniformly kappa expressing, supporting the presence of a clonal B-cell population. Additional flow cytometry studies confirmed that the population lacked expression of CD5 and CD10 (data not shown). **C** A bone marrow aspirate prior to treatment is shown, ×60 objective magnification. The granulocyte to erythroid ratio was increased by differential count (> 15:1). Residual collections of erythroid precursors showed maturational arrest, with early erythroid precursors (labeled E, corresponding to pronormoblasts). Late erythroid precursors (polychromatophilic and orhochromatic normoblasts) were not identified. **D** A post treatment bone marrow aspirate is shown, ×60 objective magnification, in contrast to the pre-treatment sample, a full spectrum of maturing erythroid precursors was now present, highlighted by abundant late erythroid precursors (labeled L)

After 5 months of receiving combined olaparib and pembrolizumab he had a good serologic and radiographic response to treatment. However, he was also noted to have progressive hypoproliferative (absolute reticulocyte count 5.6 × 10^9^ [Reference 27–120 × 10^9^]) normocytic anemia (hemoglobin (Hb) nadir of 6.5 g/dL [Reference 13.5–17.5 g/dL] prior to first transfusion in November 2019). He had no evidence of bleeding or hemolysis at the time of his initial nadir. During the course of his anemia evaluation, he developed evidence of intravascular hemolysis, with elevated lactate dehydrogenase (LDH) 1290 U/L [Reference 338–610 U/L], undetectable haptoglobin [Reference 30–200 mg/dL] and a positive Coombs test, but no thrombocytopenia or leukopenia. Olaparib (due to concerns for myelosuppression) and pembrolizumab [due to concerns for immunotherapy-induced autoimmune hemolytic anemia (AIHA)] were stopped in December 2019. Throughout his anemia evaluation, his main symptom was fatigue. Due to ongoing transfusion dependence and no improvement in his absolute reticulocyte counts, he underwent a bone marrow biopsy in February 2020 (Fig. 1). The patient’s additional past medical, drug and social history was non-contributory. His physical examination at the point of bone marrow examination revealed no evidence of lymphadenopathy or splenomegaly.

**Fig. 2:**
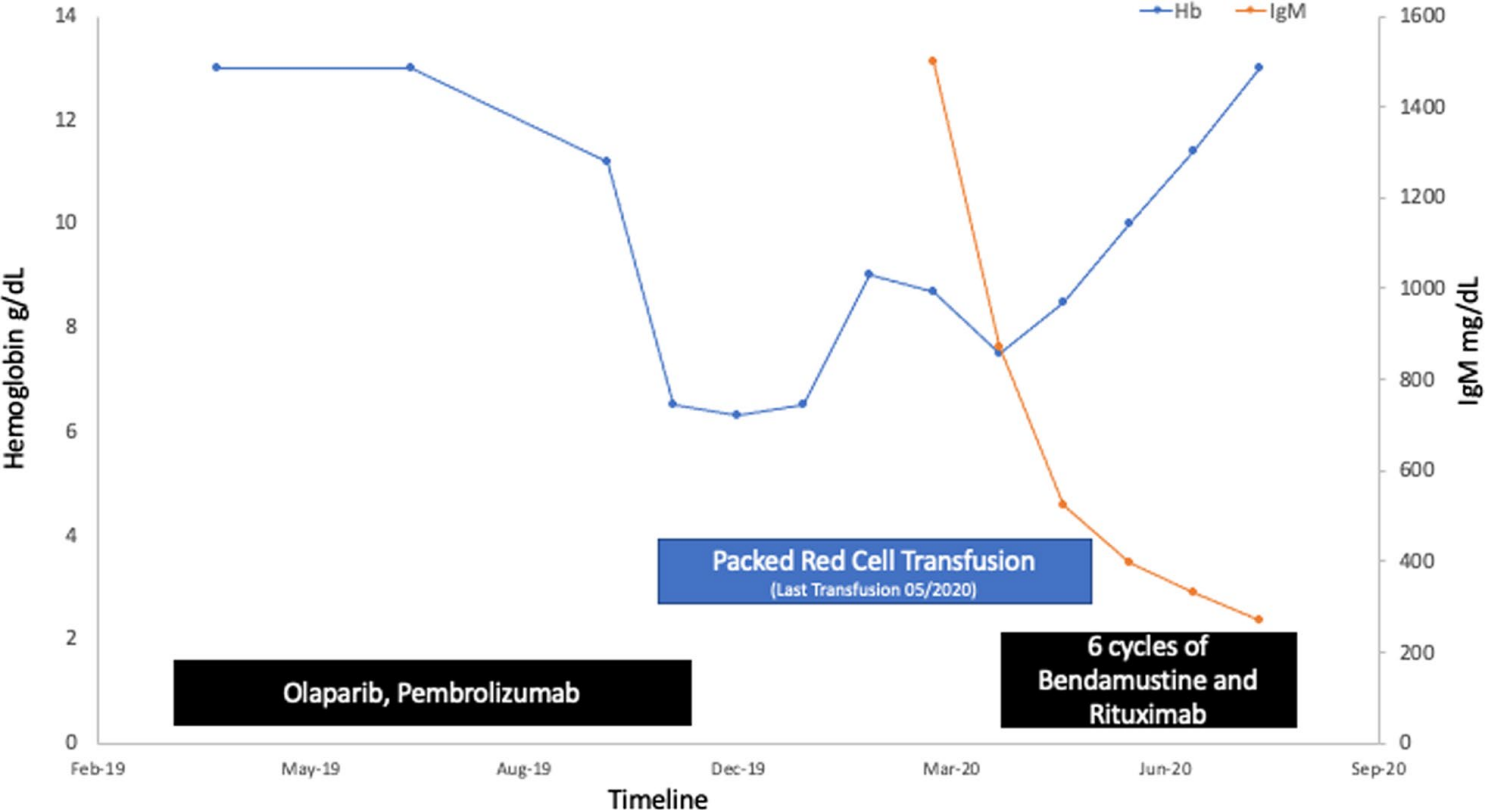
Timeline of anemia, transfusion and interventions. The patient was transfusion dependent for the management of his normocytic hypoproliferative anemia between November 2019 and May 2020. His olaparib and pembrolizumab given for the management of his prostate cancer was stopped in December 2019. A bone marrow biopsy in February 2020 revealed evidence of lymphoplasmacytic lymphoma with a *Myd88* mutation. His serum protein electrophoresis was notable for an IgM monoclonal protein. He was started on bendamustine (90 mg/m^2^ on days 1 and 2) and rituximab (500 mg/m^2^ on day 1) for 6 cycles. He was soon transfusion independent and repeat bone marrow biopsy on completion of bendamustine and rituximab demonstrated a complete response. *Hb* hemoglobin, *Ig* immunoglobulin

The notable findings on his initial bone marrow and peripheral blood evaluation were (1) CD5-negative, CD10-negative monotypic B-cell population (present in both bone marrow and peripheral blood), (2) next-generation sequencing panel with *MYD88* L265P mutation, (3) serum protein electrophoresis and immunofixation demonstrating a monoclonal IgM (kappa) paraprotein, (IgM 1499 mg/dL [Reference 35–290 mg/dL]), and (4) near absence of maturing erythroid forms noted on bone marrow and a negative parvovirus B19 serology. A multi-disciplinary discussion on the possible etiologies favored a diagnosis of lymphoplasmacytic lymphoma (LPL)/Waldenström macroglobulinemia type-IgM monoclonal gammopathy of undetermined significance (MGUS) with secondary pure red cell aplasia. He was started on bendamustine and rituximab (BR) based on the phase III findings by Rummel *et al.* [3] The patient achieved a complete response following 6 cycles of BR and his hemoglobin trends and treatment timeline are highlighted in Fig. 2. Notably, he received no further blood transfusions following cycle 3 of BR and his repeat BM biopsy demonstrated no morphologic or immunophenotypic evidence of lymphoma. In addition, his bone marrow showed trilineage hematopoiesis, including erythropoiesis with a full spectrum of maturing forms (Fig. 1). For his prostate cancer, he was continued on leuprolide alone with good disease control, however he developed brain metastases, with pathology demonstrating poorly differentiated adenocarcinoma from another unknown primary site. He received further CNS directed therapies, but he passed 3-months after completing his treatment for LPL.

## Discussion

Normocytic anemia with marked reticulocytopenia is a characteristic peripheral blood finding in patients with acquired pure red cell aplasia (PRCA) [4]. Although PRCA is an uncommon autoimmune complication seen in patients with lymphoproliferative disorders, an association with Waldenström macroglobulinemia (WM) is well described [5, 6]. The novelty of our case is the diagnosis of secondary PRCA following the introduction of pembrolizumab. WM is a rare mature B cell lymphoma found predominantly in Caucasian males, with a median age of 69 at diagnosis [7]. Most patients with WM present with anemia and interestingly in an institutional series of 12 patients with WM, 58% were identified with concomitant autoimmune disorder [6]. The identification and association of the somatic *MYD88* L265P mutations and WM was initially described in 2012 [8], and similar to our case helps provide greater diagnostic certainty when attempting to differentiate between alternative B-cell disorders. MYD88 functions as a key adaptor molecule in innate immune signaling via toll like receptors (TLRs) [8], and there is increasing recognition of the common signaling pathways involving nuclear factor-κB (NF-κB) for both WM and TLRs associated autoimmunity [9].

Attempting to rationalize the mechanisms, particularly the role played by exposure to ICI in driving the anemia in our patient was essential for therapeutic decision making. Immune related adverse events (irAE) associated with ICI is thought to be largely a T-cell mediated phenomenon [10], whereas both T-cell and B-cell processes have been implicated in the development of PRCA from underlying lymphoproliferative disorders [11]. From an epidemiologic perspective, Davis *et al.* utilized a large pharmacovigilance database to identify 168 individual case safety reports to highlight a range of hematologic toxicities that can be encountered with ICI [12] The majority of patients (*N* = 68 (40%)) had a diagnosis of autoimmune hemolytic anemia (AIHA) and 4.2% (*N* = 7) were diagnosed with PRCA [12]. The median time from ICI to hematologic adverse event was 40 days (range 3–400 days) and notably 23% of patients had an additional non-hematologic irAE reported. Most case reports with PRCA attributed to ICI have utilized steroids [12–14], with additional use of calcineurin inhibitors or immunoglobulins in steroid refractory cases [14–16].

Conversely, in a national cohort of 185 patients with PRCA, Hirokawa *et al.* describe 8 patients that have underlying lymphoma. The timeline of developing PRCA varied considerably, as did the underlying histological subtype of lymphoma [17]. In an accompanying literature search the authors demonstrated the high proportion of patients with secondary PRCA that, similar to our case, are Coombs positive [6 of 12 (50%)], and the efficacy of lymphoma directed therapies in managing the PRCA [17]. Given the clinical presentation and the clear evidence of a monoclonal B-cell population in the bone marrow and the somatic *MYD88* mutation, we attributed the PRCA to the underlying WM, which had likely been subclinical until ICI precipitated the clinical manifestation of PRCA. We, thus, assessed response following treatment directed towards his WM.

The clinical trajectory of patients with WM is highly varied and the decision to initiate therapy in addition to the choice of therapy, needs to be individualized [7]. In our patient the need to initiate treatment was relatively straightforward given his transfusion dependent symptomatic anemia. The therapeutic options were between with either fixed duration bendamustine and rituximab (BR) [3] or continuous use of ibrutinib with rituximab [18]. With the need for ongoing palliative treatment for his mCRPC, we opted for fixed duration of BR given the 91.4% response rate and median progression free survival of 78 months with this approach [3].

## Conclusion

The diagnosis of secondary PRCA in a patient receiving immune checkpoint blockade poses a diagnostic and therapeutic challenge. With increasing approvals of ICI for a variety of indications, these complicated scenarios are likely to be encountered with greater frequency. Our case highlights the importance of a systematic multidisciplinary approach, avoiding the propensity for anchoring bias on the pathophysiologic role played by ICI exposure. The identification of a concomitant diagnosis of WM in our patient with mCRPC treated with ICI vastly changed the management plan, with lymphoma directed therapies highly efficacious in treating both the underlying indolent lymphoma and the associated secondary PRCA. The limitation is that the nature of a retrospective report precludes the causal role of ICI in PRCA.

## Data Availability

All data are contained in this report.
